# Kali Bichromicum

**Published:** 1884-06

**Authors:** J. T. Kent

**Affiliations:** St. Louis


### KALI BICHROMICUM.

PROFESSOR J. T. KENT, A. M., M. D., ST. LOUIS.

The following symptoms have recently been cured by Kali bichrom. They are found under Kali bic. in *Allen's Encyclopædia of Pure Materia Medica*, page 237:

Weakness of digestion, so that the stomach was disordered by any but the mildest food (chrome washers). Incarceration of flatulence in stomach and whole lower portion of abdomen. (Zlatarovich.)

Great feebleness of stomach in the morning. (Lackner.)

Feeling of emptiness in the stomach, though want of appetite, at dinner. (Marenzetler.)

Feeling of sinking in the stomach before breakfast. (Dr. R. Dudgeon.)

The patient wakes in the night with great uneasiness in the stomach, and soreness and tenderness in a small spot to the left of the xephoid appendix, which is very similar to symptoms in Drysdale's proving.

Sudden violent pain in the stomach, in its anterior surface, a burning constrictive pain. (Zlatarovich.)

The same patient complained of repletion after a mouthful of food, and he had taken Lycopodium without benefit.

There was also cutting as with knives, and he was unable to digest potatoes or any starchy food.

There were no catarrhal symptoms of nose or chest, and no thick, ropy, mucous discharges, therefore Kali bich. was neglected. The stomach symptoms alone guided to its use, as he had no other symptoms of importance.

The relief is marked, and I think permanent.

It will be seen that I have made use of the language of the prover mostly, as it so perfectly describes the symptoms of the patients.

In looking over the proving, the patient underscored such symptoms as he had suffered from, and the remedy was furnished on these symptoms, which really lends value to the provings. Especially are these provings the more beautiful, as they are by several provers.

